# Confounding Factor Analysis for Vocal Fold Oscillations

**DOI:** 10.3390/e25121577

**Published:** 2023-11-23

**Authors:** Deniz Gençağa

**Affiliations:** 1 Department of Electrical and Electronics Engineering, Antalya Bilim University, Antalya 07190, Turkey; d.gencaga@ieee.org; 2 Language Technologies Institute, School of Computer Science, Carnegie Mellon University, Pittsburgh, PA 15213, USA

**Keywords:** speech processing, information theory, confounding factor analysis, transfer entropy

## Abstract

This paper provides a methodology to better understand the relationships between different aspects of vocal fold motion, which are used as features in machine learning-based approaches for detecting respiratory infections from voice recordings. The relationships are derived through a joint multivariate analysis of the vocal fold oscillations of speakers. Specifically, the multivariate setting explores the displacements and velocities of the left and right vocal folds derived from recordings of five extended vowel sounds for each speaker (/aa/, /iy/, /ey/, /uw/, and /ow/). In this multivariate setting, the differences between the bivariate and conditional interactions are analyzed by information-theoretic quantities based on transfer entropy. Incorporation of the conditional quantities reveals information regarding the confounding factors that can influence the statistical interactions among other pairs of variables. This is demonstrated on a vector autoregressive process where the analytical derivations can be carried out. As a proof of concept, the methodology is applied on a clinically curated dataset of COVID-19. The findings suggest that the interaction between the vocal fold oscillations can change according to individuals and presence of any respiratory infection, such as COVID-19. The results are important in the sense that the proposed approach can be utilized to determine the selection of appropriate features as a supplementary or early detection tool in voice-based diagnostics in future studies.

## 1. Introduction

In a complex system with many variables and parameters, e.g., a system comprising a set of coupled high-dimensional partial differential equations with no closed-form solution, the analysis of dependencies and interactions between its components is a very challenging task. Such systems arise in many areas of science and engineering, such as environmental sciences [1,2], machine learning and data science [3,4], epidemiology and public health [5], finance [6], speech pathology, otolaryngology, vocal fold biomechanics [7], astrophysics and celestial mechanics [8], quantum mechanics [9], financial modeling [10], biological systems [11], material science and engineering [12], and social network modeling [13]. Generally, high-dimensional models are used to explain or predict the behavior of complex real-life processes and phenomena. In such cases, the numerical solutions of the models are studied and used for most practical purposes such as prediction or gauging the long-term effects of various hypothetical changes. Due to the importance of such models in multiple fields, methodologies that allow us to understand the complex interactions between the various components of their multidimensional outcomes are extremely important. This paper presents a methodology that can reveal some of these unknown interactions among the variables of a complex system from data.

For decades, the traditional practice has been to examine and characterize these interactions by means of classical statistical approaches based on correlation analysis [14]. However, it is widely known that correlation analysis cannot efficiently discover nonlinear interactions and/or higher-order statistical properties [15,16]. It is also not adequate to reveal the complex relationships among different variables. In the literature, these shortcomings have been mitigated using information-theoretic approaches [17] that use entropy and other quantities, such as mutual information and transfer entropy [18,19,20].

In information theory, mutual information (MI) is a measure of the mutual dependence between two variables and it can be expressed in terms of joint and marginal Shannon entropies. It quantifies the “amount of information” obtained about one random variable by observing the other random variable. Mutual information is a special case of a more general quantity called relative entropy, which is a measure of the distance between two probability distributions. Entropy quantifies the expected “amount of information” held in a random variable and it is also a measure of the uncertainty of a random variable [18]. MI is a symmetric quantity and it does not reveal any information regarding the direction of the interaction between the variables. Its definition in terms of the probability density functions (PDFs) allows us to not only analyze second-order statistical properties, but also other higher-order interactions between any two variables.

Transfer entropy (TE), unlike MI, is an asymmetric quantity and provides the direction of information flow between two variables. “Information flow” is a term used to indicate how the uncertainty of observing a random variable from its own samples changes by the observation of the samples of another variable. In other words, it informs the affecting and the affected variables in the case of the presence of such an interaction. Since the work of Schreiber [19], TE has been actively utilized in many fields to understand the directional interactions between random variables [20].

While MI and TE can help us understand the interaction between variables, the problem is that they are defined between *two* variables, and are not sufficient to simultaneously analyze the interactions among multiple variables. The lack of theoretical approaches to generalize these information-theoretic quantities in multi-variable settings has led to other approaches for multi-variable information processing, such as Partial Information Decomposition (PID) and Interaction Information (II) [21,22,23]. In these approaches, concepts like synergy and redundancy are also defined.

This paper provides a statistical methodology, called Confounding Factor Analysis for Vocal Fold Oscillations (CFA-VFO), to investigate multivariate interactions among the variables of the voice production system (the process of phonation) in humans. Here, the phonation process is characterized by the Vocal Fold Oscillation (VFO) model. The VFO model is composed of a set of differential equations where the expected motion of the vocal folds of a speaker is produced as a solution. The solution set of this model is obtained as time series representing the displacements and velocities of left and right vocal folds, and it is provided by the Adjoint Least Squares (ADLES) algorithm, which is proposed in [24]. The CFA-VFO is based on the estimation of the confounding factors, affecting the bivariate interactions among different pairs of variables. This is achieved by the analysis of the differences between the conditional TE and bivariate TE values (Transfer Entropy Difference or TED) estimated from data. The simulations show how CFA-VFO can be used to better understand how each of these variables affects the others, especially by bringing out the non-obvious dependencies between these variables within and across the two vocal folds. The results demonstrate the conditional interactions between these variables and reveal the confounding variables that affect the directional interactions between them. Most importantly, it is demonstrated that this multi-variable approach can reveal important details about real-life contexts, including people, sounds, and disease conditions from recorded speech, while the bivariate analysis between the variables is insufficient for this.

Recently, there have been studies exploring the potential for detecting respiratory infections, including COVID-19, through the analysis of vocal phonation or speech in voice-based diagnostics. While it is important to note that vocal analysis is not a primary method for COVID-19 diagnosis, it has been investigated as a supplementary or early detection tool. Some studies have found that changes in vocal patterns can be associated with respiratory illnesses, including COVID-19 [25,26,27,28,29,30].

In the following section, the background of the VFO model and its relevant details are presented, followed by the explanation of the proposed methodology. The details of the proposed methodology are demonstrated on an analytically tractable model in Section 4. Following this demonstration, a proof of concept is presented in the experiments, where a curated, clinically validated dataset of the recorded voices of patients affected with COVID-19 are analyzed. In the last section, the results are discussed and directions for future research are outlined.

## 2. The VFO Model and the Effect of COVID-19

Phonation is the process whereby we produce voiced sounds during speech production. During this process, the vocal folds vibrate in a self-sustained fashion, driven by the balance of aerodynamic forces across the glottis. The process is mediated by the muscles of the larynx that control the vocal folds, but it is not in itself sustained by these muscles. The muscles only play a role in starting, modulating, and stopping the phonation process during speech production [31].

In recent years, physical models that can emulate the motion of the vocal folds during phonation have been used to deduce the actual motion of the vocal folds [24,31,32]. In one approach called the Adjoint Least-Squares (ADLES) algorithm [24], the parameters of the chosen model are estimated from the recorded speech signal by comparing it to an estimated glottal flow waveform using an inverse filtering procedure, and following an iterative optimization process. Once the parameters are estimated in this manner, the model is numerically solved to obtain the time series corresponding to its variables, namely the displacements, and velocities of both vocal folds of the speaker during phonation.

**Figure 1:**
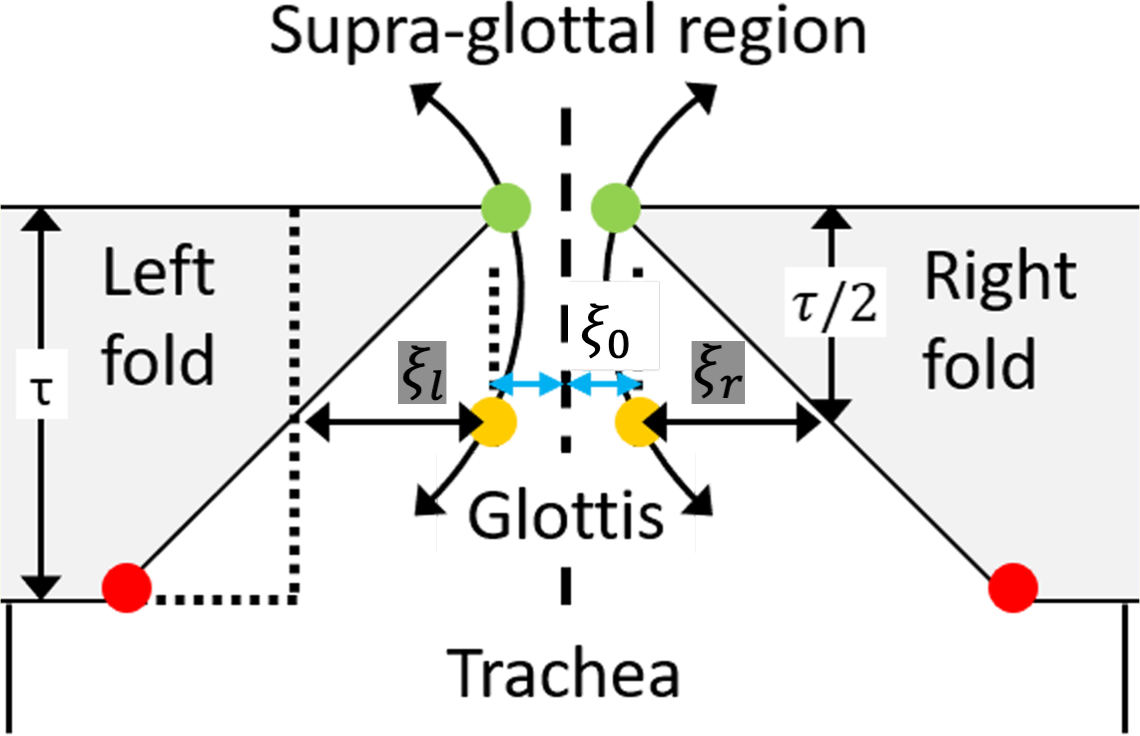
Diagram of the one-mass body-cover model for vocal folds. The lateral displacements at the midpoint of the left and right vocal folds are denoted as $\xi_{l}$ and $\xi_{r}$, and $\xi_{0}$ represents the half glottal width at rest [25].

In this paper, the VFO model in [24] is used, where a specific formulation for the one-mass asymmetric model from [32] is adopted. A schematic diagram of the basic components of this model is given in Figure 1. In this figure, the centerline of the glottis is denoted as the *z*-axis. Displacements are measured with respect to the midpoint of the vocal folds, for which z=0, and the left and right vocal folds oscillate with lateral displacement $\xi_{l}$ and $\xi_{r}$, resulting in a pair of coupled Van der Pol oscillators [33,34], where each oscillator is a non-conservative system with non-linear damping. The coupled oscillators evolve in time according to the second-order differential equation given as follows:

(1) $$\begin{array}{cc} & {\ddot{\xi }}_{r}+\beta \left(1+\xi_{r}^{2}\right){\dot{\xi }}_{r}+\xi_{r}-\frac{\Delta }{2}\xi_{r}=\alpha \left({\dot{\xi }}_{r}+{\dot{\xi }}_{l}\right)\\ & {\ddot{\xi }}_{l}+\beta \left(1+\xi_{l}^{2}\right){\dot{\xi }}_{l}+\xi_{l}+\frac{\Delta }{2}\xi_{l}=\alpha \left({\dot{\xi }}_{r}+{\dot{\xi }}_{l}\right) \end{array}$$

where β is the coefficient incorporating mass, spring, and damping coefficients, α is the glottal pressure coupling coefficient, and Δ is the asymmetry coefficient. For a male adult with normal voice, the reference values for the model parameters (from clinical measurements) are usually approximately set to α=0.5, β=0.32, and Δ=0 [24,35]. The overall algorithm for solving this, using the ADLES method, is mentioned in [24] and described step-by-step in detail in [35]. On implementing the algorithm and numerically solving the dynamical system in Equation (1), the time series corresponding to $\xi_{l},\xi_{r}$ and $\dot{\xi_{l}},\dot{\xi_{r}}$ are obtained. These are the four variables that are analyzed by using the procedure described in the following section. It is important to note that the dependencies among the time series corresponding to these variables are not evident or predictable from the dynamical system alone, since closed-form solutions do not exist.

In the literature, the same VFO model has been used in modeling the vocal fold oscillations to detect the presence of COVID-19 infection [25]. To achieve this goal, the solution of the VFO model is obtained from two groups of data, which are voice recordings consisting of people with and without COVID-19, respectively. Later, the phase space trajectories for the left and right vocal folds for COVID-19-positive and negative individuals are illustrated and a distinct pattern difference is noticed between the two groups. It is shown that even simple classifiers can yield high detection accuracies using the recordings of isolated extended vowels as a result.

## 3. Multivariate Dependencies: Confounding Factor Analysis

Statistical interactions between variables, such as those of the VFO model, have traditionally been analyzed using correlation analysis. Traditional correlation analysis (also known as Pearson’s correlation) is defined between two random variables *X* and *Y* as follows:

(2) $$\rho_{X,Y}=\mathrm{corr}\left(X,Y\right)=\frac{\mathrm{cov}(X,Y)}{\sigma_{X}\sigma_{Y}}=\frac{E\left[\left(X-\mu_{X}\right)\left(Y-\mu_{Y}\right)\right]}{\sigma_{X}\sigma_{Y}},\;\mathrm{if}\;\sigma_{X}\sigma_{Y}>0$$

where E[.] denotes expectation, cov(.) stands for covariance, $\mu_{X}$ and $\mu_{Y}$ denote the expectations and $\sigma_{X}$ and $\sigma_{Y}$ denote the standard deviations of variables *X* and *Y* [14].

Correlation analysis suffers from a few drawbacks. The correlation coefficient is by definition a measure of the strength of the linear relationship between two variables. It ranges from −1 to 1, where −1 indicates a perfect negative linear relationship, 1 indicates a perfect positive linear relationship, and 0 indicates no linear relationship. However, the correlation coefficient is limited in its ability to detect nonlinear relationships between variables.

The inability of correlation to detect nonlinear relationships arises from its reliance on assumptions of linearity. The Pearson correlation coefficient is calculated using the covariance between the two variables, divided by the product of their standard deviations. This calculation assumes that the relationship between the variables is linear, which means that the change in one variable is proportional to the change in the other variable, and so it does not (by virtue of this limiting assumption) capture complex, nonlinear associations. As a fallout of this, correlation analysis becomes sensitive to outliers, which means the correlation coefficient is influenced by the extreme values of data points that deviate significantly from the general trend of the data, leading to an overestimation or underestimation of the strength of the linear relationship.

Nonlinear relationships can take various forms, such as exponential, logarithmic, or polynomial relationships. The Pearson correlation coefficient can fail to detect these relationships, since it is only designed to measure linear relationships. To analyze nonlinear relationships, alternative methods such as Spearman’s rank correlation, Kendall’s tau, or nonlinear regression techniques are considered [15]. However, they each have their own limitations, and they primarily focus on pairwise relationships between two variables.

Spearman’s rank correlation measures the strength of the monotonic relationship between two variables, which is a broader category than just linear relationships. It does not extend to relationships involving more than two variables. On the other hand, it has some serious drawbacks. For example, it can be sensitive to tied ranks, which occur when two or more data points have the same value. This can lead to biased estimates of the correlation, particularly in small sample sizes. It is also a non-parametric method, meaning that it does not rely on any assumptions about the underlying distribution of the data. While this can be advantageous, it can also result in lower statistical explainability compared to parametric methods, especially when the data follow a normal distribution.

Kendall’s tau measures the strength and direction of the ordinal association between two variables, making it useful for detecting both linear and nonlinear monotonic relationships. However, like Spearman’s rank correlation, it is primarily designed for pairwise relationships between two variables. Similar to Spearman’s rank correlation, Kendall’s tau is sensitive to tied ranks, which can lead to biased estimates of the correlation. It can also be computationally intensive, especially for large datasets, as it requires comparing every pair of data points.

To analyze relationships involving more than two variables, techniques such as multivariate regression (requiring models), partial correlation, or multivariate dependence measures like canonical correlation analysis (CCA) can be employed. CCA is a multivariate statistical technique that primarily operates as a linear method. It seeks to identify linear combinations of variables in two datasets in such a way that the correlation between these linear combinations is maximized [36]. CCA assumes a linear relationship between the variables within each dataset and between the two datasets themselves. If the relationships between variables are highly nonlinear, CCA may not capture those nonlinear dependencies effectively.

Recently, “causality” has become one of the most frequently studied topics in machine learning [37]. Causality refers to the relationship in which one event, process, state, or object (referred to as a cause) plays a role in generating another event, process, state, or object (an effect), where the cause is partially accountable for the effect, and the effect is somewhat reliant on the cause [38]. One of the fundamental concepts in causality can be expressed through “confounding factors”. A confounding factor is a variable that influences both the dependent variable and independent variable, causing a spurious association. Confounding cannot be described in terms of correlations [38]. CFA aims to identify and control for variables that may distort the relationship between an exposure (independent variable) and an outcome (dependent variable) in observational studies. In the literature, statistical techniques like multiple regression, stratification [39], or propensity score matching [40] are used in CFA to adjust for the influence of confounding variables. The goal is to obtain an unbiased estimate of the causal relationship between the exposure and the outcome while controlling for potential confounders. One of the approaches to detect confounding has been recently proposed by [41], by using spectral analysis.

The basic difference between confounding factor analysis (CFA) and canonical correlation analysis (CCA) is that the former focuses on identifying and controlling for confounding variables to obtain unbiased estimates of causal relationships, while canonical correlation analysis aims to explore linear relationships between two sets of variables by finding linear combinations with the highest correlation. These methods serve different purposes and use distinct methodologies to address their respective objectives.

In the next section, the details of the proposed CFA-VFO method are presented. This is another approach to confounding factor analysis, where information-theoretic quantities are utilized.

### An Information-Theoretic Approach to Confounding Factor Analysis

In this section, the proposed CFA approach is presented in detail with its background material. The method is based on the analysis of the bivariate and conditional TE values between each pair of variables in the system. For the sake of completeness, the main information-theoretic quantities are defined first. To define “information”, Shannon entropy is widely preferred in the literature, which is defined as follows for discrete random variables:

(3) $$H\left(X\right)=-\sum_{x\in X}p\left(x\right)\log p\left(x\right)$$

where p(x) denotes the probability density function of the variable. Shannon entropy is thus the average uncertainty for finding the system at a particular state *x* out of a possible set of states *X*.

Given two datasets denoted by *X* and *Y*, the mutual information (MI) between them can be written as follows:

(4) $$MI\left(X;Y\right)=\sum_{x\in X}\sum_{y\in Y}p\left(x,y\right)\log\frac{p(x,y)}{p(x)p(y)}$$

where the MI represents the divergence between the joint distribution p(x,y) of variables *x* and *y* and the product p(x)p(y) of the two marginal distributions. The MI is a symmetric quantity (MI(X;Y)=MI(Y;X)) and can be rewritten as a sum and difference of Shannon entropies, as seen below:

(5) MI(X;Y)=H(X)+H(Y)−H(X,Y)

where MI(X;Y) represents the mutual information between variables *X* and *Y*, H(X) represents the Shannon entropy of variable *X*, H(Y) represents the Shannon entropy of variable *Y*, and H(X,Y) represents the joint entropy of variables *X* and *Y* [18].

Transfer entropy (TE) can be thought of as an extension of MI where the directed interaction is analyzed between two Markov processes [19]. TE quantifies the degree to which the past values of random process *Y* help predict the future values of the other random process *X*, beyond what can be predicted from the past values of *X* alone. Here, *X* and *Y* denote the random variables that represent the instantaneous values of their corresponding random processes at a certain time. In a similar way, TE can be estimated to predict the future values of *Y* from *X*. If one of these processes is affecting the other, meaning that the other is affected, the interaction between these two processes is said to be directed in one way. This means that this kind of interaction depends on the affecting (source) and the affected processes (target) and it is also called an asymmetric relationship. In other words, the information is said to flow from the source to the target.

The TE from *Y* to *X* is defined by the following equation:

(6) $$TE_{YX}=\sum p\left(x_{t+1},x_{t},y_{t}\right)\log\frac{p(x_{t+1}|x_{t},y_{t})}{p(x_{t+1}|x_{t})}$$

where:

- $TE_{YX}$ represents the transfer entropy from variable *Y* to variable *X*.
- $x_{t}$ and $x_{t+1}$ are the values of variable *X* at time *t* and time t+1, respectively.
- $y_{t}$ is the value of variable *Y* at time *t*.
- $p(x_{t+1},x_{t},y_{t})$ is the joint probability distribution of $x_{t+1},x_{t}$, and $y_{t}$.
- $p(x_{t+1}|x_{t},y_{t})$ is the conditional probability distribution of $x_{t+1}$ given $x_{t}$ and $y_{t}$.
- $p(x_{t+1}|x_{t})$ is the conditional probability distribution of $x_{t+1}$ given $x_{t}$.

Unlike MI, transfer entropy is a directed measure. The information flow from *Y* to *X* ($TE_{YX}$) is different from the information flow from *X* to *Y* ($TE_{XY}$), which is defined below:

(7) $$TE_{XY}=\sum p\left(y_{t+1},y_{t},x_{t}\right)\log\frac{p(y_{t+1}|y_{t},x_{t})}{p(y_{t+1}|y_{t})}$$

In the literature, “information flow” terminology is frequently used to address the difference between TE values (i.e., the information flow is said to be from variable *X* to variable *Y*, if $TE_{XY}$ is greater than $TE_{YX}$). Both MI and TE are capable of detecting linear and nonlinear relationships between variables, making it very useful for analyzing complex systems. This is due to the definition of these information-theoretic quantities where probabilistic distributions are exploited, unlike the limited statistical properties (first- and second-order moments) involved in correlation.

In a more general format, given two variables, TE is the amount of uncertainty reduced in future values of a random process by knowing the past values of another process given past values of itself. Thus, the asymmetry of TE results in a differentiation of the two directions of information flow. This is demonstrated in the following equation that defines the transfer entropy directed from *X* to *Y*:

(8) $$\begin{array}{cc} TE_{XY} & =T\left(Y_{i+1}|Y_{i}^{\left(k\right)},X_{i}^{\left(l\right)}\right)\\ & =\sum_{y_{i+1},y_{i}^{\left(k\right)},x_{i}^{\left(l\right)}}p\left(y_{i+1},y_{i}^{\left(k\right)},x_{i}^{\left(l\right)}\right)\log\frac{p\left(y_{i+1}|y_{i}^{\left(k\right)},x_{i}^{\left(l\right)}\right)}{p\left(y_{i+1}|y_{i}^{\left(k\right)}\right)} \end{array}$$

The equation below defines the transfer entropy directed from *Y* to *X*:

(9) $$\begin{array}{cc} TE_{YX} & =T\left(X_{i+1}|X_{i}^{\left(k\right)},Y_{i}^{\left(l\right)}\right)\\ & =\sum_{x_{i+1},x_{i}^{\left(k\right)}y_{i}^{\left(l\right)}}p\left(x_{i+1},x_{i}^{\left(k\right)},y_{i}^{\left(l\right)}\right)\log\frac{p\left(x_{i+1}|x_{i}^{\left(k\right)},y_{i}^{\left(l\right)}\right)}{p\left(x_{i+1}|x_{i}^{\left(k\right)}\right)} \end{array}$$

where $x_{i}^{\left(k\right)}=\left\{x_{i},\ldots ,x_{i-k+1}\right\}$ and $y_{i}^{\left(l\right)}=\left\{y_{i},\ldots ,y_{i-l+1}\right\}$ are past states, and *X* and *Y* are $k^{th}$- and $l^{th}$-order Markov processes, respectively, such that *X* depends on *k* previous values of itself and *l* previous values of *Y*. In the literature, *k* and *l* are also known as the embedding dimensions. In Equations (6) and (7), the case of *l* = *k* = 1 is considered. TE analysis has been a key tool in the research field of causality due to its asymmetric directional structure.

In this paper, the TE definitions given above will be called *bivariate TE*, as they are defined between two variables *X* and *Y*. Clearly, TE in its current bivariate form cannot be used to detect any possible effect of a third variable on this interaction. To better understand such multi-variable interactions, additional information-theoretic quantities are required. To analyze the probable effects of a third variable on the interactions between *X* and *Y*, the conditional TE based on a third variable *Z* is introduced and compared with bivariate TE. This third variable is known as a confounding factor in statistics and constitutes one of the most important research sub-fields in the analysis of causality. To analyze these conditionalities, conditional TE quantities are defined as follows:

(10) $$\begin{array}{cc} TE_{XY|Z} & =T\left(Y_{i+1}|Y_{i}^{\left(k\right)},X_{i}^{\left(l\right)},Z_{i}\right)\\ & =\sum_{y_{i+1},y_{i}^{\left(k\right)},x_{i}^{\left(i\right)},z_{i}}p\left(y_{i+1},y_{i}^{\left(k\right)},x_{i}^{\left(l\right)},z_{i}\right)\log\frac{p\left(y_{i+1}|y_{i}^{\left(k\right)},x_{i}^{\left(l\right)},z_{i}\right)}{p\left(y_{i+1}|y_{i}^{\left(k\right)},z_{i}\right)} \end{array}$$

(11) $$\begin{array}{cc} TE_{YX|Z} & =T\left(X_{i+1}|X_{i}^{\left(k\right)},Y_{i}^{\left(l\right)},Z_{i}\right)\\ & =\sum_{x_{i+1},x_{i}^{\left(k\right)},y_{i}^{\left(l\right)},z_{i}}p\left(x_{i+1},x_{i}^{\left(k\right)},y_{i}^{\left(l\right)},z_{i}\right)\log\frac{p\left(x_{i+1}|x_{i}^{\left(k\right)},y_{i}^{\left(l\right)},z_{i}\right)}{p\left(x_{i+1}|x_{i}^{\left(k\right)},z_{i}\right)} \end{array}$$

Here, to identify the confounding factors, the analysis of the differences between the bivariate and conditional TE values is proposed. As a variant of CFA, these differences, which are called TE differences (TEDs), are defined and formulated as follows:

(12) $$TED_{XYZ}=TE_{XY}-TE_{XY|Z}$$

(13) $$TED_{YXZ}=TE_{YX}-TE_{YX|Z}$$

**Figure 2:**
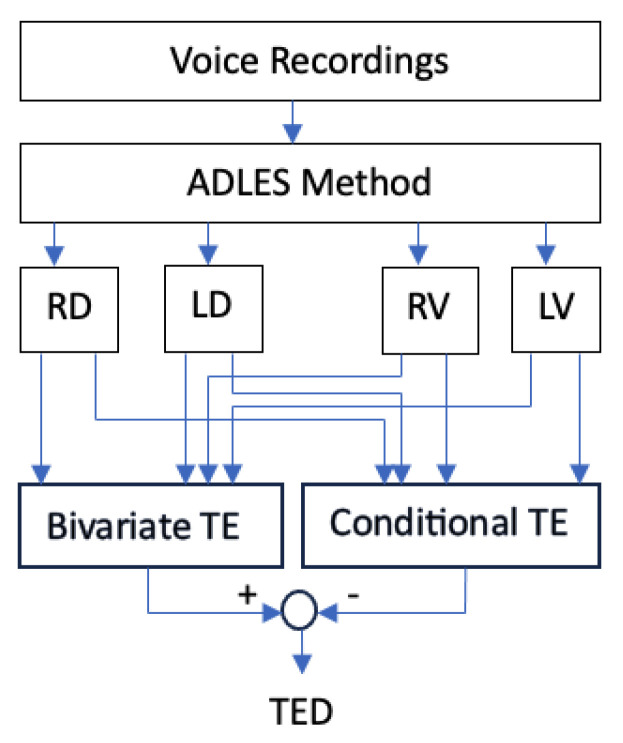
Flow chart of the CFA-VFO approach. Here, RD, LD, RV, and LV stand for the right displacement, the left displacement, the right velocity, and the left velocity variables, respectively.

In Figure 2, a brief block scheme is given to visualize the proposed CFA-VFO approach:

In the next section, the proposed confounding factor analysis approach is first applied to a first-order vector autoregressive system of three Gaussian distributed variables. Under these assumptions, the analytical expressions of the proposed methodology are obtained. Later, as a proof of concept, this methodology is utilized to analyze the effects of the confounding factors to better understand the interactions between the variables of the VFO model in Equation (1) under the presence of a respiratory infection, such as COVID-19.

## 4. Confounding Factor Analysis on an Analytically Tractable Model

In signal processing, autoregressive processes are widely utilized to model signals which are correlated in time. If the statistical interactions need to be modeled among more than one autoregressive process, the couplings between them can be incorporated into a vector matrix form, also known as a vector autoregressive model (VAR) [42]. A VAR process of order *K* is represented by VAR(*K*) and this model can be expressed in the following form:

(14) $$y_{t}=\Phi_{1}y_{t-1}+\Phi_{2}y_{t-2}+\cdots +\Phi_{K}y_{t-K}+n_{t}$$

where $y_{t}={\left[y_{1,t},y_{2,t},\ldots ,y_{d_{1},t}\right]}^{T},n_{t}={\left[n_{1,t},n_{2,t},\ldots ,n_{d_{1},t}\right]}^{T}$ and $\Phi_{j}$ denote the VAR(K) process, driving noise and $\left(d_{1}\times d_{1}\right)$ matrix of AR coefficients for j=1,2,…,K, respectively.

In this section, the following VAR(1) system is analyzed to better understand and demonstrate the confounding factor effects:

(15) $$v_{t}=Gv_{t-1}+n_{t}$$

(16) $$\left[\begin{array}{c} x_{t}\\ y_{t}\\ z_{t} \end{array}\right]=\left[\begin{array}{ccc} 0.99 & 0 & 0\\ 0.5 & 0.8 & 0\\ 0.2 & 0.3 & 0.7 \end{array}\right]\left[\begin{array}{c} x_{t-1}\\ y_{t-1}\\ z_{t-1} \end{array}\right]+\left[\begin{array}{c} n_{1,t}\\ n_{2,t}\\ n_{3,t} \end{array}\right]$$

where $E\left[n_{t}n_{t}^{T}\right]=\Sigma_{n}$ denotes the covariance matrix of $n_{t}$, which is taken to be a three-dimensional identity matrix with diagonal values equal to 0.01. Here, samples of $n_{t}$ are identical and independently distributed Gaussian variables. The TE calculations of such linear systems driven by Gaussian distributed processes can be expressed in closed form. The general bivariate and conditional TE expressions are previously given by Equation (6) through Equation (11). In this example, the embedding dimensions are taken to be *l* = *k* = 1, leading to the use of the reduced equations given in Equations (6) and (7). It is known that the pdf and the conditional pdf of variables with Gaussian distributions can be analytically expressed. If the pdf terms in Equations (6) and (7) are substituted by their Gaussian expressions, the bivariate TE equations can be given by the following closed-form expressions:

(17) $$TE_{YX}=\frac{1}{2}\log\left[\frac{\det\left(\Sigma_{x_{i+1}|x_{i}}\right)}{\det\left(\Sigma_{x_{i+1}|x_{i},y_{i}}\right)}\right]=\frac{1}{2}\log\left[\frac{\det\left(\Sigma_{x_{i+1},x_{i}}\right)\cdot \det\left(\Sigma_{x_{i},y_{i}}\right)}{\det\left(\Sigma_{x_{i+1},x_{i},y_{i}}\right)\cdot \det\left(\Sigma_{x_{i}}\right)}\right]$$

and

(18) $$TE_{XY}=\frac{1}{2}\log\left[\frac{\det\left(\Sigma_{y_{i+1},y_{i}}\right)\cdot \det\left(\Sigma_{y_{i},x_{i}}\right)}{\det\left(\Sigma_{y_{i+1},y_{i},x_{i}}\right)\cdot \det\left(\Sigma_{y_{i}}\right)}\right]$$

where Σ shows the following covariance matrix:

(19) $$\Sigma =\mathrm{cov}\left(\begin{array}{c} x_{i}\\ y_{i}\\ z_{i}\\ x_{i+1}\\ y_{i+1}\\ z_{i+1} \end{array}\right).$$

This covariance matrix is composed of 36 elements. In Equations (17) and (18), sub-block matrices of this covariance matrix are shown by using the relevant indices. For example, $\Sigma_{x_{i+1},x_{i},y_{i}}$ denotes the 3 × 3 sub-block matrix of Σ, where the first, second, and fifth rows and columns are taken. Similarly, the other sub-block matrices can easily be formed by taking the corresponding rows and columns of Σ [43]. To analyze the confounding factor effect in the system given by Equation (16), the following closed-form expressions of conditional TE can be used in the case of Gaussian distributed processes:

(20) $$TE_{YZ|X}=\frac{1}{2}\log\left[\frac{\det\left(\Sigma_{z_{i+1}|z_{i},x_{i}}\right)}{\det\left(\Sigma_{z_{i+1}|z_{i},x_{i},y_{i}}\right)}\right]=\frac{1}{2}\log\left[\frac{\det\left(\Sigma_{z_{i+1},z_{i},x_{i}}\right)\cdot \det\left(\Sigma_{z_{i},x_{i},y_{i}}\right)}{\det\left(\Sigma_{z_{i},x_{i}}\right)\cdot \det\left(\Sigma_{z_{i+1},z_{i},x_{i},y_{i}}\right)}\right]$$

and

(21) $$TE_{ZY|X}=\frac{1}{2}\log\left[\frac{\det\left(\Sigma_{y_{i+1}|y_{i},x_{i}}\right)}{\det\left(\Sigma_{y_{i+1}|y_{i},x_{i},z_{i}}\right)}\right]=\frac{1}{2}\log\left[\frac{\det\left(\Sigma_{y_{i+1},y_{i},x_{i}}\right)\cdot \det\left(\Sigma_{z_{i},x_{i},y_{i}}\right)}{\det\left(\Sigma_{x_{i},y_{i}}\right)\cdot \det\left(\Sigma_{y_{i+1},y_{i},x_{i},z_{i}}\right)}\right].$$

**Figure 3:**
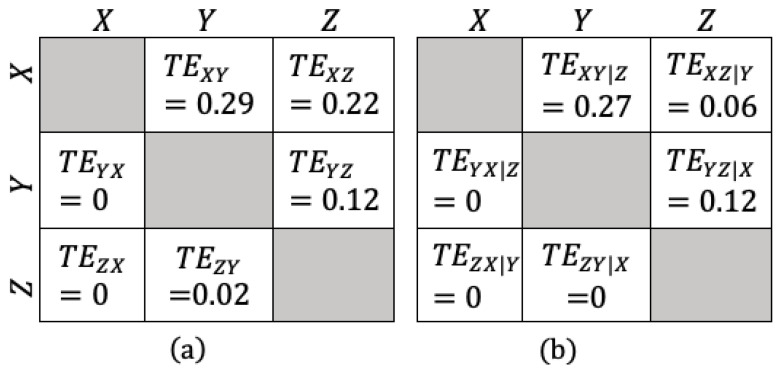
Analytical values of the TE values between the variables of the VAR(1) example. (**a**) Bivariate TE, (**b**) conditional TE results.

For the VAR system in Equation (16), the TE values are provided in Figure 3.

In Equation (16), the interactions between the variables are chosen to be from *X* to *Y* and from both *X* and *Y* to *Z*. These interactions are verified by the bivariate TE values. For example, it is seen that the interaction direction (information flow) from *X* to *Y* is 0.29 nats, whereas it is zero in the opposite direction, leading to the conclusion that the net flow is from *X* to *Y*, as expected. Moreover, in Equation (16), variable *X* affects both *Y* and *Z*, which makes it a *confounding factor* affecting the interaction between *Y* and *Z*. This effect cannot be inferred by using bivariate TE analysis alone. Therefore, it is clear that the proposed approach in this paper is informative to catch this kind of multi-variable interactions. When the conditional TE values are examined, it is seen that the magnitude of the interaction between *Y* and *Z* is changed when the effect of the underlying confounding factor *X* is considered as a conditionality.

## 5. Proof of Concept: Experiments

In this section, a potential application of the proposed methodology is presented. The proposed approach is used to analyze the changes in the interactions of the vocal fold oscillations during a respiratory infection. Here, transfer entropy differences as defined in Equations (12) and (13) are utilized to study and compare the vocal fold oscillations of healthy speakers and speakers affected with COVID-19. It is important to emphasize that this problem setting was only chosen as an illustrative case, and is not expected to be a diagnostic aid for COVID-19 in the real-world case. For the latter to happen, large volumes of speech signals from thousands of speakers need to be analyzed. Here, the goal is to demonstrate the potential use of the proposed TED-based approach.

The dataset used in the demonstration was collected under clinical supervision and it was curated by a private company in Chile. The dataset included recordings from 512 individuals who were tested for COVID-19 and turned out to be either positive or negative following the clinically administered PCR tests. Among these, the recordings from eighteen individuals were selected. This group was composed of nine infected and nine healthy individuals. The speech signals were sampled at 8 kHz, and recorded over microphones on commodity devices. From each speaker, five different extended vowel sounds were collected, which are given as follows: /aa/ as in aardvark, /iy/ an in tree, /ey/ as in slate, /ow/ as in grow, and /uw/ as in blue.

For the application, four time series are used for each speaker. These signals represent the vocal fold oscillations of the corresponding speakers in uttering the corresponding sounds. These four signals, which are called the Right Vocal Fold Displacement (RD), Right Vocal Fold Velocity (RV), Left Vocal Fold Displacement (LD), and Left Vocal Fold Velocity (LV), are obtained by the ADLES algorithm. After the time series corresponding to these four variables are obtained, bivariate and conditional TE values are calculated, as depicted in Figure 2.

In the literature, different methods have been proposed to accurately estimate TE values from data [20,44]. The main difference of these approaches lies in the approximation of the probability density functions. Histograms, multivariate Gaussian models, kernel density estimates, and the Kraskov method are some of the most frequently used methods in the TE literature [44]. In this paper, the Gaussian model-based approach is used to estimate the bivariate and conditional TE values from data. For this purpose, an open-source toolkit (JIDT [44]) is utilized. In this tool, the statistical significance of the estimations is reported via *p*-values. During the simulations, the threshold for significance level is set to 0.05 and the insignificance of any estimate is represented by *NaN (Not a Number)*.

### Observations

In the experiments, the four variables representing RD, RV, LD, and LV are studied in pairs (corresponding to *X* and *Y* in the equations above) in different permutations, and they are also conditioned on a third variable, one at a time for the TED analysis.

**Figure 4:**
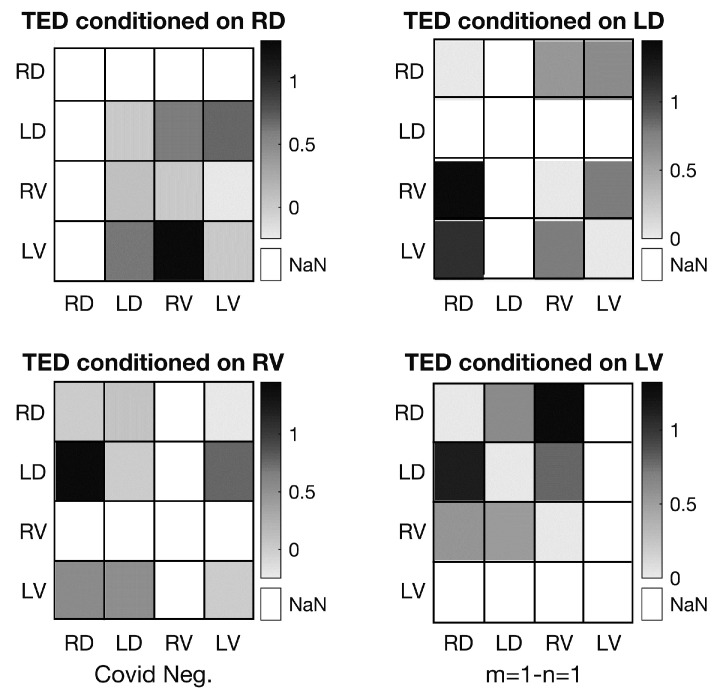
TED values for a person with negative COVID-19 measurements.

For each pair, the values of $TED_{XY}$ and $TED_{YX}$ are coded on a matrix for visualization. An example is shown in Figure 4, which is presented to help clarify and understand this representation for the reader.

Figure 4 shows the results of the TED analysis on the voice of a COVID-negative person, uttering one of the sounds from the five-phoneme set. Here, each one of the four matrices uses one of the four variables as the conditioning variable *Z* in Equations (12) and (13). In each matrix, each row corresponds to the source variable for the information flow, and each column corresponds to the destination variable for the information flow.

Given a conditioning variable, the row and column corresponding to the same variable are not meaningful or relevant in this study. For example, in the upper left matrix, the source variable RD in the first row and the destination variable RD in the first column are the same as the conditioned variable, which is also RD. So, in this study, these portions of the matrix are not meaningful, as they are conditioned on themselves. Therefore, they are undefined, and they are also represented in the figure with *NaN* (Not a Number). The rest of the cells in each matrix remain meaningful for this analysis.

The significance of transfer entropy being low versus high lies in the degree of directed information transfer between two variables. As explained in the previous sections, transfer entropy measures the extent to which past values of one variable (*Y*) help predict the future values of another variable (*X*), beyond what can be predicted from the past values of *X* alone. When the transfer entropy from variable *Y* to variable *X* is low, it indicates that the past values of *Y* do not significantly contribute to predicting the future values of *X*. In other words, there is little or no directional information flow from *Y* to *X*. This could mean that the two variables are either independent, or that any dependencies between them are weak or causally irrelevant. For the TED values to be low, the TE in the first terms of Equations (12) and (13) must not be significantly higher compared to the corresponding second terms, regardless of the absolute values of the terms.

This means that when TED is observed to be low, we have a situation where it does not make much of a difference to the directional information flow from *Y* to *X*, even if it is conditioned on *Z*. In such cases, *Z* exerts little or no influence on *X*, and *X* and *Y* are relatively independent of *Z*.

On the other hand, when the TE from variable *Y* to variable *X* is high, it suggests that the past values of *Y* help predict the future values of *X* to a considerable extent, beyond what can be predicted from the past values of *X* alone. This implies a strong directional information flow from *Y* to *X*, and *possibly a causal relationship between the variables*. It is important to note here that TE itself is a relative measure and does not have an absolute scale. Comparing absolute TE values across different pairs of variables has no clear meaning unless the physics of the situation can be used to throw some light on such comparisons.

When the TED values are observed to be high, the variable *Z* serves to confound the dependencies between *Y* and *X*, lowering the predictability of *X* from past values of *Y* (hence the nomenclature “confounding variable” for *Z*).

Keeping these points in mind, the following statements can be inferred from the valid cells in the top left matrix of Figure 4:

1. Information flow from LV to RV is most confounded by RD, while the exact opposite holds for information flow from RV to LV, i.e., it is the most independent of RD. This implies that the activity of the right vocal fold exerts a strong influence on the velocity of the left vocal fold, while the reverse is not true. This reveals a surprising **unilateral influence** of one vocal fold on the other (influence of R on L).
2. Information flow from LV to LV, RV to RV, and LD to LD is hardly affected, and is independent of RD, as expected.
3. Information flow from LV to LD, and the reverse, LD to LV, and also more significantly LD to RV, is similarly confounded by RD. This is expected if the “**unilateral influence**” hypothesis is valid. There is no doubt that velocity and displacement are physically highly correlated, but can be affected by mass.

As a specific example, in the top left matrix of Figure 4, the TED value from LD to RV is shown, corresponding to its value of 0.59 nats. As TED is the difference between the bivariate TE and the conditional TE, this value indicates that after the observation of RD, the bivariate TE from LD to RV is increased by 0.59 nats, indicating that extra information is gained as a result of this conditioning. Thus, it can be concluded that the left vocal fold displacement affects right vocal fold velocity and this (the degree of the influence) is dependent on right vocal displacement, which acts as a confounding factor affecting both variables, in the background.

As a result of this analysis performed on all of the matrices, it can be concluded that one vocal fold actually seems to influence and dominate the motion of the other for this particular normal speaker. This may be highlighting something with biological significance in the process of voice production, such as a connection to the right-handedness or left-handedness of the person whose speech is analyzed. However, such biological conclusions require additional support via clinical procedures. Beyond the motions of the vocal folds and the physics of the used model, the biological aspects of the proposed approach are out of the scope of this work. Here, the main focus is on the theoretical development of the confounding factor analysis methodology, which is believed to be a very promising tool to have more in-depth knowledge about the unknown interactions.

**Figure 5:**
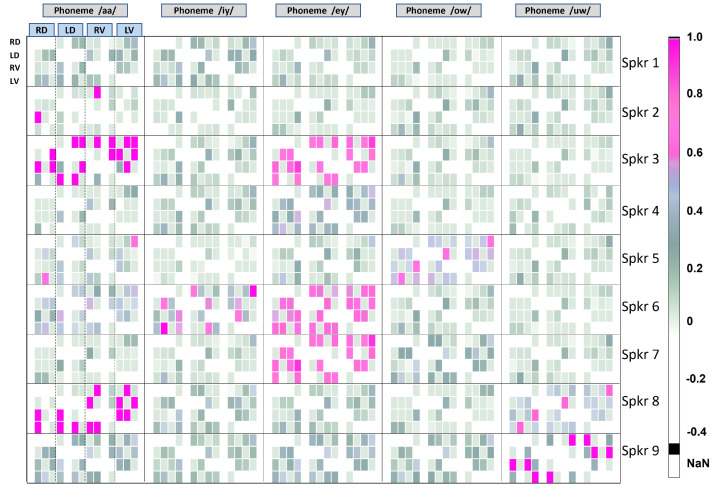
TED values for nine people who tested negative for COVID-19. Vocal fold oscillation dynamics are analyzed for five extended vowel sounds uttered by each person.

In Figure 5, the TED values for all pairs and confounding factors are given for nine people who tested clinically negative for COVID-19. Sets of four rows, counted from the top, correspond to each speaker. Each speaker uttered five different phonemes, and the analysis has therefore been performed for each phoneme in sequence. The phoneme identities are given at the top of the figure. For each phoneme and each speaker, sixteen columns are shown. From the left, each set of four columns is conditioned by the variable labeled at the top in the blue box to obtain the corresponding TED values. The order of the variables for the columns (not shown in the figure due to space issues) is the same as the order of the rows in each set of four: RD, LD, RV, and LV, repeating for each conditioning variable shown in the corresponding blue box above.

**Figure 6:**
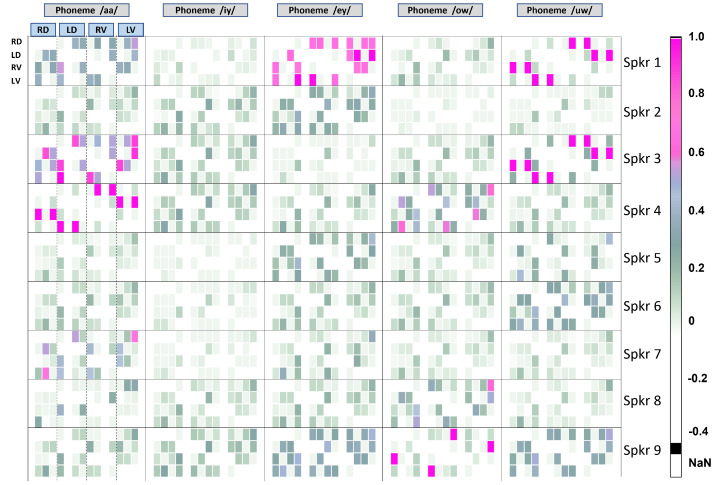
TED Values for nine people who tested positive for COVID-19. Vocal fold oscillation dynamics are analyzed for five extended vowel sounds uttered by each person.

The same configuration of rows and columns is shown for the data from COVID-19-positive speakers in Figure 6. The speakers in Figure 5 and Figure 6 are *not the same*. Although this could be avoided in a longitudinal study with more data, it is considered as a future project where the main scope will be the detection of COVID-19 or similar infections from vocal data by using CFA. It should be noted that this study proposes a statistical tool to analyze such biological phenomena. Nevertheless, some interesting and important insights can be drawn from Figure 5 and Figure 6. These are listed below.

From Figure 5 and Figure 6, we note the following:

1. TED values are in general higher for normal people, as compared to those affected by COVID-19. This supports the known fact that in normal, non-pathological cases of voice production, the vocal folds act in synchrony and are strongly coupled and highly entrained. Thus, their displacements and velocities are expected to be well-correlated and inter-related, and not easily confounded by other influencing factors.
2. Conversely, TED values are in general lower for affected people, indicating that the vocal folds are less entrained, less predictable, and more susceptible to confounding influences.
3. There is no canonical pattern of dependencies across individuals, regardless of their health status. Every individual’s vocal fold oscillations are different. It remains to be seen if they are also unique. However, this can only be revealed through studies performed on very large populations in future projects where the CFA-VFO approach will be utilized as a tool.
4. For normal people, the phonemes /aa/, /ey/, and /uw/ show more propensity for being influenced by confounding factors.
5. For the phoneme /ey/, three speakers out of nine in the normal group show almost the same pattern of TEDs. This indicates the presence of some factor of biological significance that could be related to some common articulatory-phonetic characteristic of this phoneme. The phoneme /ey/ is a diphthong, which means it is a complex voiced sound that consists of two distinct vowel qualities within the same syllable. It is an oral vowel, so the velum (soft palate) is raised, preventing airflow through the nasal cavity. In the case of /ey/, the sound begins with an open-mid front unrounded vowel and moves towards a close-mid front unrounded vowel. From an articulatory-phonetic perspective, to produce the phoneme /ey/, the tongue starts in a relatively low (open-mid) and front position in the oral cavity for the first vowel quality, which is similar to the position for /e/ as in “Bet”. As the sound progresses, the tongue moves upward and slightly forward towards a close-mid front position, similar to the position for /ih/ as in “Bit”. Throughout the production of /ey/, the lips remains unrounded. The corners of the lips may be slightly tensed or spread. The vocal tract remains relatively open during the production of /ey/, with the oral cavity taking on a more front-focused resonance due to the front position of the tongue. Perhaps it is the articulatory-phonetic complexity of /ey/ that requires people to adhere to more common vocal patterns. This is at least a hypothesis that can be made given the surprising degree of commonality in the TED patterns.
6. Example of a finer-level observation: For speaker 3 (normal case), the information flow from LV to RD and RV is mostly confounded by LD. It is only minimally affected by other confounding variables. For this speaker, the left vocal fold “influences” the right vocal fold in uttering the phone /aa/. This is not the case with other speakers. This could potentially help to identify the speaker, if it bears out across a large number of speaker and phone-specific recordings.
7. In the case of COVID-19-positive individuals, generally, there are fewer confounding factors in evidence. This indicates more loose coupling between different variables during phonation.
8. For the vowel sound /uw/, information flow patterns are similar across multiple pairs of individuals. Some aspect of articulation seems to be at play for this sound, making it more difficult for COVID-affected people to produce this sound. The phoneme /uw/ is a voiced monophthong, which means it is a simple vowel sound consisting of a single, steady vowel. It is a close back rounded vowel. During its production, the tongue is positioned high (close) and towards the back of the oral cavity. The lips are rounded and protruded (a characteristic feature of close back vowels). In addition, the vocal tract is relatively constricted due to the high position of the tongue, and the oral cavity takes on a more back-focused resonance due to the back position of the tongue. This may make it relatively more difficult for people affected by a respiratory condition to individualize the sound. Again, this is only a hypothesis that this analysis makes possible. Its validity remains to be properly proved or disproved by biological studies, which are out of the scope of this work.

These are a few examples of the conclusions of TED analysis in the setting of voice production in normal and pathological cases. Many finer-level observations are possible, and can be tested for validity in larger data and analysis settings.

## 6. Conclusions

In this paper, a novel approach known as Transfer Entropy Difference (TED) is introduced to assess the impact of a specific variable on the statistical interactions between two other variables. TED is presented as a valuable tool, enabling a deeper understanding of how variables within complex systems interact beyond conventional pairwise relationships. Traditional methods rely on measures like the correlation coefficient, mutual information, and bivariate transfer entropy to analyze these relationships. However, TED offers a more nuanced perspective by exploring the predictability and information flow between variables and how they are influenced by intervening third variables. The methodology is elucidated using an analytically tractable vector autoregressive system, incorporating a confounding factor that affects the interaction between the other two variables. The study underscores the inadequacy of conventional bivariate transfer entropy analysis in identifying such interactions and emphasizes the necessity of TED. Importantly, the proposed TED-based confounding factor analysis proves its ability to extract detailed insights into variable interactions, supported by analytically tractable, closed-form mathematical expressions.

Following these methodological advancements, the paper demonstrates the practical application of TED in a case study. Voice recordings from eighteen individuals in two distinct groups (infected and non-infected) are examined to deduce vocal fold oscillations from the recorded speech. The TED analysis of left and right vocal fold displacements and velocities reveals potentially crucial biological information, surpassing the capabilities of traditional pairwise techniques. This multivariate approach provides a comprehensive analysis of confounding factors influencing pairwise interactions between left and right displacement and velocity variables. Specifically, within the context of voice production and phonation, the experiment illustrates TED’s effectiveness in illuminating the underlying physics of the modeled process. The study anticipates that with sufficient data, TED analysis can generate discriminative features for machine learning-based automated systems, facilitating predictions or classifications in complex phenomena. Future work aims to apply this promising approach to more extensive datasets and present a detailed statistical performance analysis.

## Data Availability

The data presented in this study are available on request from the corresponding author.
